# Mitral regurgitation in the critically ill: the devil is in the detail

**DOI:** 10.1186/s13613-023-01163-4

**Published:** 2023-08-02

**Authors:** Chris F. Duncan, Emma Bowcock, Faraz Pathan, Sam R. Orde

**Affiliations:** 1 Department of Intensive Care Medicine, Nepean Hospital, Kingswood, Sydney, NSW 2747 Australia; 2 Department of Cardiology, Nepean Hospital, Kingswood, Sydney, NSW 2747 Australia; 3 Nepean Clinical School of Medicine, Charles Perkin Centre Nepean, University of Sydney, Kingswood, Sydney, NSW 2747 Australia; 4 University of Sydney, Camperdown, Sydney, NSW 2006 Australia

## Abstract

Mitral regurgitation (MR) is common in the critically unwell and encompasses a heterogenous group of conditions with diverging therapeutic strategies. MR may present acutely with haemodynamic instability or more insidiously with failure to wean from mechanical ventilation. Critical illness is associated with marked physiological stress and haemodynamic changes that dynamically influence the severity and implication of MR. The expanding role of critical care echocardiography uniquely positions the intensivist to apply advanced bedside valvular assessment to recognise haemodynanically significant MR, manipulate and optimise cardiopulmonary physiology and identify patients requiring urgent cardiology and surgical referral. This review will consider common clinical scenarios, therapeutic strategies and the pearls and pitfalls of echocardiographic assessment and quantification in the critically unwell.

**Supplementary Information:**

The online version contains supplementary material available at 10.1186/s13613-023-01163-4.

## Pearls:

- Detection of mitral regurgitation (MR) has significant treatment implications in the critically ill and is important not to miss.
- A high level of suspicion and early echocardiography is key.
- Acute, severe MR may be challenging to diagnose both with clinical examination and with echocardiography. A hyperdynamic left ventricle (LV) in the context of shock should prompt evaluation for life threatening acute severe MR.
- In significant MR, a higher LV ejection fraction (EF) is expected. An EF of < 60% is abnormal and represents LV decompensation.
- Explaining the mechanism is equally as important as grading severity.
- Don’t be deceived by eccentric MR jets. A systematic approach is needed using all ultrasound modalities: 2D, colour, pulse wave Doppler (PWD) and continuous wave Doppler (CWD).

## Introduction

Mitral regurgitation (MR) is commonly seen in the critically ill and recognition of haemodynamically significant MR using echocardiography (echo) is essential for tailoring management strategies. Issues arise as standard guidelines for the assessment and quantification of MR using echo are derived from the non-critical care population with stable and more predictable physiology [6–8]. The intensive care specialist is uniquely positioned to integrate advanced bedside valvular assessment to influence supportive therapy, prevent or recognise decompensation and guide early referral for definitive intervention [1]. Accordingly, comprehensive valvular assessment is an important facet of advanced critical care echocardiography.

MR may present acutely with cardiovascular instability and respiratory failure, or more insidiously as failure to wean from mechanical ventilation [2, 3]. Critical illness is associated with physiological stress and haemodynamic changes that dynamically influence the severity and implication of MR: adrenergic stimulation, catecholamines, positive pressure ventilation and heart–lung interactions all have the potential to affect MR severity [4–6]. Critical illness frequently tips the scales to favour mitral regurgitation into the left atrium over effective forward stroke volume (SV), leading to a declining spiral of worsening shock and left atrial/pulmonary venous hypertension.

We will discuss common clinical scenarios, the pearls and pitfalls of echo assessment and quantification of MR in critically ill patients.

## Mitral valve anatomy

**Fig. 1:**
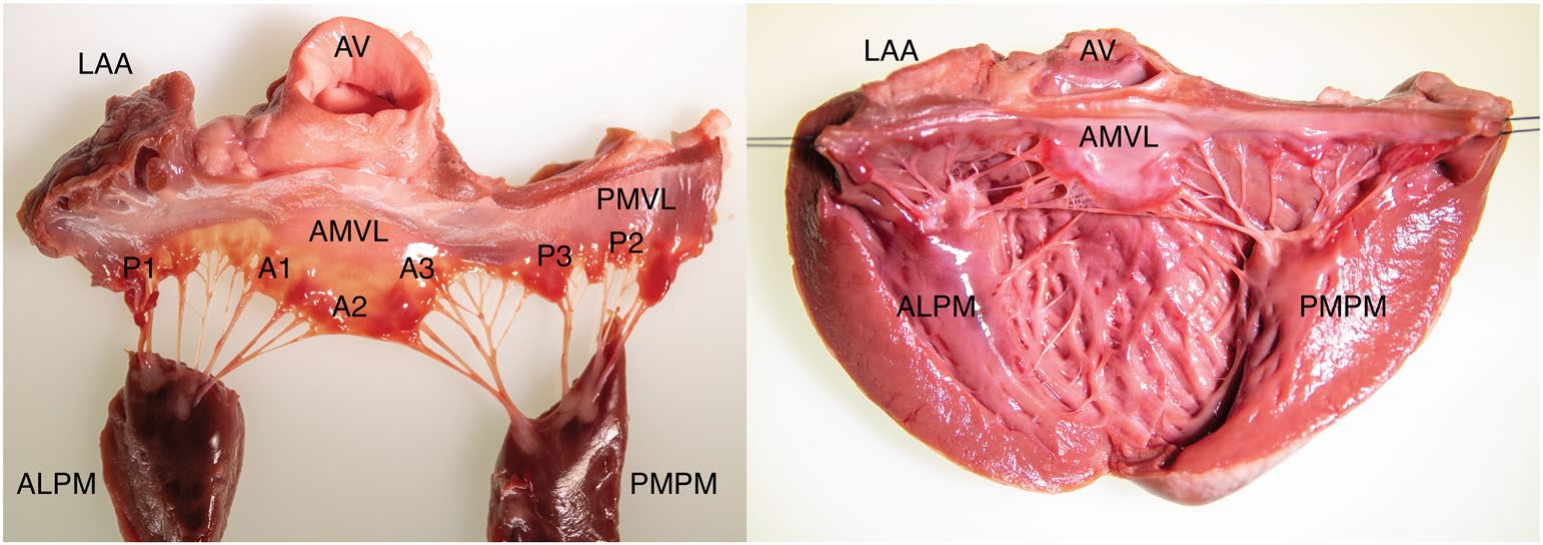
Mitral valve anatomy**.** Mitral valve anatomy and adjacent structures. Note that the anterior mitral valve leaflet does not possess scallops and its close proximity to the aortic valve. Each papillary muscle provides chordae tendineae to both valve leaflets. ALPM, anterolateral papillary muscle; PMPM, posteromedial papillary muscle; LAA, left atrial appendage; AV, aortic valve; AMVL, anterior mitral valve leaflet; PMVL, posterior mitral valve leaflet

Any significant mitral regurgitation requires anatomical explanation**.** Disruption of any component of the valvular or subvalvular apparatus can lead to significant regurgitation. The saddle shaped mitral annulus forms part of the fibrous skeleton of the heart (Fig. 1). The mitral annulus provides an anchor for two asymmetrical valve leaflets—the anterior and posterior mitral valve leaflets (AMVL and PMVL, respectively). The AMVL inserts into the anterior one-third of the annulus but is longer than the PMVL and thus occupies two-thirds of the total mitral valve area. The PMVL is a ‘C’ shaped structure with indentations partitioning the valve into distinct scallops—P1, P2 and P3 [7]. Although the AMVL does not possess scallops, conventional nomenclature subdivides the valve into A1, A2 and A3 regions to correspond with its neighbouring PMVL [7]. A1 and P1 are the most anterolateral scallops located adjacent to the left atrial appendage. The valvular free edges meet at the coaptation zone where they overlap to provide a seal during ventricular systole. At the margins of the coaptation zone the AMVL and PMVL join to form the anterolateral and posteromedial commissures.

Two papillary muscles, the anterolateral and posteromedial papillary muscles, provide chordae tendinae to *both* *valve* *leaflets* (Fig. 1). The single blood supply of the posteromedial papillary muscle by the posterior descending artery (typically a branch of the right coronary artery; less frequently the circumflex artery) renders it susceptible to ischaemia and rupture in the context of an inferior myocardial infarction. The anterolateral papillary muscle receives dual blood supply (left anterior descending and circumflex arteries) and is consequently less prone to ischaemia [8, 9].

## Mechanism, mechanism, mechanism

Any significant mitral regurgitation requires *mechanistic* *explanation* (e.g. ruptured chordae, poor coaptation from annular dilation, etc.). MR may be categorised as *acute* *versus* *chronic*, *primary* *versus* *secondary* or using the *Carpentier* classification [10–12].

### Acute versus chronic MR

Chronic MR should be considered in those with a history of mitral regurgitation or with risk factors such as hypertension, ischaemic heart disease, cardiomyopathy or renal failure [13]. MR leads to increased LA volume, increased LV preload and reduced forward stroke volume (SV) [14–16]. In response, the LV is remodelled through dilatation and eccentric hypertrophy with an aim to preserve forward SV and normalise afterload/wall stress [15, 16]. Importantly, despite progressive deterioration in LV contractile function, ejection fraction (EF) commonly remains within the normal range [17–19]. The adaptive mechanisms of the LV, which include serial increases in myocyte sarcomeres and myofibril slippage, are complex and beyond the scope of this article. The reader is directed to previous comprehensive and dedicated reviews of LV remodelling in MR [14, 16].

In the compensated phase of MR, LA compliance and LA SV increase in response to the increased volume (operating on the ‘ascending’ limb of the Frank–Starling curve) [20, 21]. This limits the rise in LA pressure (LAP) and enhances pulmonary venous drainage [22].

With progressive volume overload and wall stress, compensatory mechanisms are overwhelmed leading to progressive LV/LA dilation, impaired function and reduced forward SV [15, 16, 21]. LA volume overload results in maladaptive processes including myocyte growth, hypertrophy, necrosis and apoptosis alongside alterations in the extracellular matrix and interstitial fibrosis [18, 21]. The rise in LV end-diastolic volume (LVEDV) and LA volume are unable to increase cardiac output and may worsen MR due to annular dilatation (operating on the ‘descending’ limb of the Frank–Starling curve) [18, 20, 21, 23]. LAP increases and LA function/compliance diminishes, reducing the protective cushioning effect of the LA and adversely impacting pulmonary haemodynamics [18, 20, 22, 24]. Patients with chronic MR are therefore at significant risk of decompensation during the physiological stress and interventions associated with critical illness and are an important group to identify early [25].

Acute pathology, e.g. myocardial infarction, papillary muscle rupture or endocarditis, may result in acute MR. The rapidity of onset renders the heart incapable of compensating for the acute volume overload of the LV and LA leading to reduced forward SV, cardiogenic shock, raised LAP and acute pulmonary oedema [2, 26]. In addition to the structural abnormality causing acute MR, LV systolic function may appear normal or hyperdynamic with an absence of anatomical compensation such as LA/LV dilation or eccentric hypertrophy [27, 28]. This emphasises the need for intensivists to contextualise LV systolic function with cardiac output measurement. The absence of structural adaptation makes acute MR challenging to detect both clinically and with echocardiography [12, 29]. The MR jet may be underestimated despite significant regurgitant volume due to reduced MR driving pressure from a combination of hypotension and raised LAP [27]. Compensatory tachycardia also shortens the time for detection. Applying multiple imaging techniques, including Doppler to detect severity and upstream/downstream consequences, and transoesophageal echocardiography (TOE) may improve the detection of acute severe MR [30].

LV volume, LA volume and patient’s antecedent history may be used to differentiate chronic from acute MR. Indexing LA volume to body surface area improves the detection of LA enlargement independent of the patient’s body size and gender [31, 32]. The absence of LA dilation almost excludes the presence of chronic, severe MR [18, 28]. However, as LA dilation is present in many other conditions, including atrial fibrillation, hypertension and diastolic dysfunction, its presence does not necessarily indicate severe MR [27].

Caution should be used to not dismiss acute on chronic MR which is commonly due to chordal rupture in a patient with moderate or severe MR [33, 34]. These cases present with acute features of pulmonary oedema and possibly haemodynamic instability with echocardiographic findings suggestive of chronic MR (e.g. LA/LV dilation) [35–37]. In such cases, reviewing previous imaging or performing TOE can help to identify new structural changes.

### Primary versus secondary MR

MR is conventionally classified as *primary*, due to structural abnormalities of the valve or subvalvular apparatus, or *secondary,* due to left ventricular or atrial disease, causing tethering of the valve leaflets (also known as *functional* MR). It is possible for a mixed primary and secondary mechanism to be present, particularly in elderly patients with degenerative or calcific mitral valve disease, although a *dominant* *mechanism* can usually be identified that becomes the focus of therapeutic strategies [38].

### Carpentier classification

Carpentier proposed a classification for MR in 1983 based upon leaflet motion to guide surgical intervention which has since been modified [10, 39]. **Type** **I** describes MR in the setting of *normal* leaflet motion that is typically centrally directed. In **type** **II**, the leaflet motion is *increased* with either mitral prolapse or flail through the coaptation line causing an eccentrically directed MR jet *away* from the affected leaflet. **Type** **III** is subdivided into **type** **IIIa** where leaflet motion is restricted during both systole and diastole (e.g. rheumatic mitral valve disease), whereas **type** **IIIb** the leaflet restriction is limited to systole causing eccentric MR *towards* the restricted leaflet. The MR jet in type III may be central or eccentric.

## MR in critical illness

**Fig. 2:**
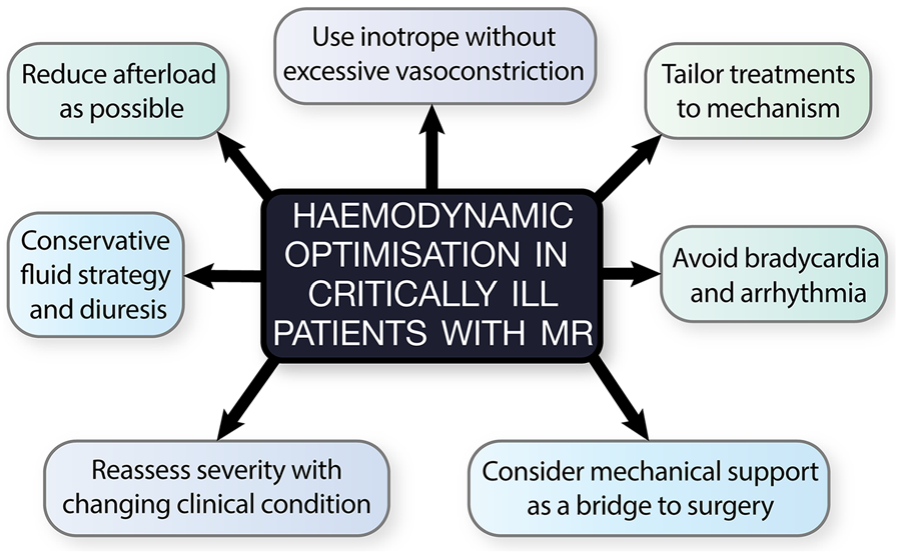
Management principles of MR in the critically ill

Certain clinical phenotypes should prompt the ICU clinician to consider the presence of haemodynamically significant MR. Whilst far from exhaustive, the following cases (Table 1) aim to emphasise the need to integrate clinical context (including loading conditions) with systematic echocardiography to evaluate the mechanism and severity of MR to direct management. The dynamic nature of MR necessitates repeat assessment after therapeutic interventions or altered loading conditions. Key principles of management are summarised in Fig. 2.

**Table 1:** Cases demonstrating clinical phenotypes

**Case 1**: A 62-year-old male presenting to ED with severe respiratory failure and shock	**Case 2**: A 67-year-old female with escalating noradrenaline and FiO2 requirements post-emergency laparotomy
2-day history of fevers and feeling generally unwell	12-h following laparotomy and bowel resection for small bowel obstruction
History of intravenous drug use (none for 3 months)	History of hypertension, type 2 diabetes mellitus and chronic kidney disease stage III
Rapid escalation in FiO2 requirement and shock in the Emergency Department requiring intubation	Progressive rise in vasopressor and FiO2 requirement with cool peripheries
Temperature 37.7°C, FiO2 0.6 (invasive mandatory ventilation), noradrenaline 0.2 mcg/kg/min, cool peripheries	Temperature 36.4°C, FiO2 0.5 at 50l flow via high flow nasal oxygenation, noradrenaline 0.4 mcg/kg/min
Mildly elevated troponin. Lactate 11	Chest X-Ray demonstrated bilateral pulmonary infiltrates

**Case 3**: A 43-year-old with productive cough, chest discomfort and unilateral pulmonary infiltrates on chest X-Ray	**Case 4**: A 76-year-old female with non-retroviral pneumocystic jirovecii pneumonia with two failed spontaneous breathing trials
24-h history of productive cough and fevers	6 day admission to ICU requiring invasive ventilation for severe respiratory failure
Evolving breathlessness since symptom onset	History of hypertension, dyslipidaemia and smoking
History of antiphospholipid syndrome with multiple pulmonary emboli and an RCA myocardial infarction treated with drug eluting stents. Mildly elevated troponin, no new ECG changes	Previous TTE revealed mild mitral regurgitation
FiO2 0.5 on CPAP 10 cmH_2_O	Two spontaneous breathing trials resulted in tachycardia, hypertension, worsening hypoxaemia and bilateral pulmonary infiltrates
Right sided coarse crackles on examination

### Clinical phenotype 1—shock with normal or hyperdynamic LV systolic function

**Fig. 3:**
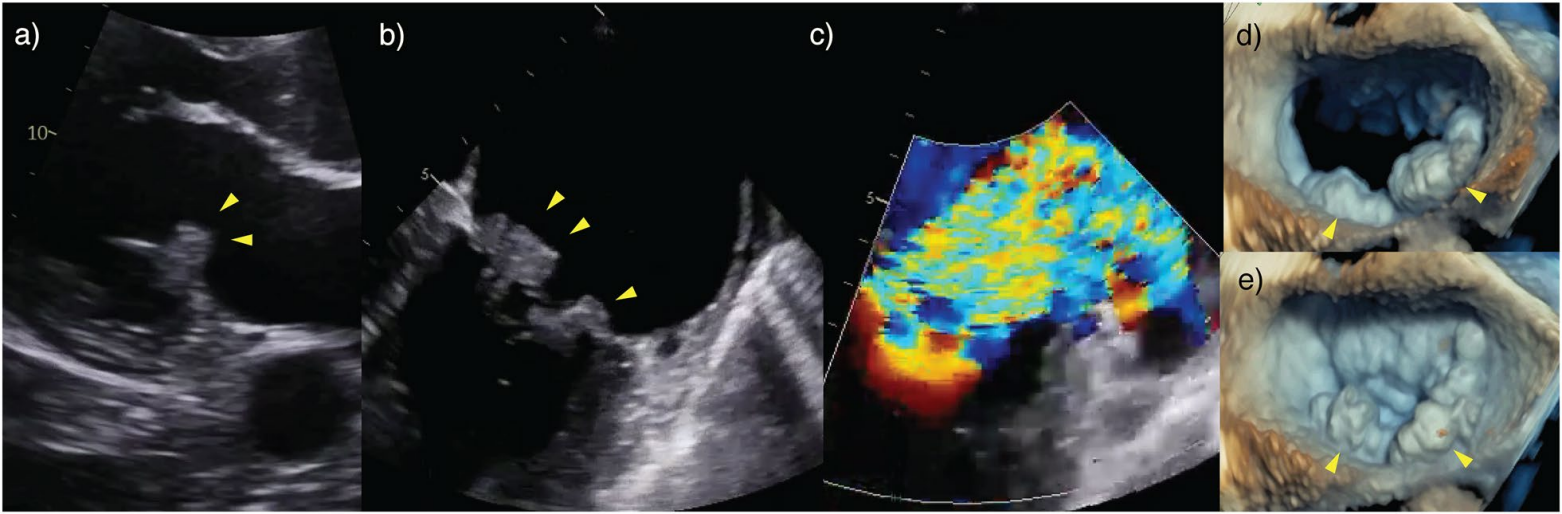
Case 1—Infective endocarditis. Vegetations highlighted with arrows on the **a**) parasternal long-axis (PLAX) TTE view and (**b**) mid-oesophageal commissural TOE view; **c** colour Doppler showing severe MR; **d** (diastole) and **e** (systole) demonstrates a 3D TOE image of the mitral valve vegetations (arrows)

Case 1 describes a case of native mitral valve endocarditis with severe/torrential MR in a patient with a history of intravenous drug use. This patient presented to the ED with scant clinical history and non-specific features of fevers, respiratory failure and shock. Initial management was guided by recommendations from the Surviving Sepsis Campaign including intravenous fluid boluses and early vasopressor therapy; both interventions that potentially exacerbate regurgitation fraction in MR [40–42]. The patient rapidly deteriorated requiring intubation, mechanical ventilation and escalating doses of noradrenaline. An urgent comprehensive TTE was performed and demonstrated mitral valve vegetations with severe MR (Fig. 3a)(Additional file 1: Figure 4 Video—Case 1—Infective Endocarditis). A TOE confirmed vegetations on P1/P2/P3 with a flail chord (Fig. 3b–e). In response to this new finding, noradrenaline was transitioned to adrenaline for enhanced inotropy and chronotropy, PEEP was increased, and an intra-aortic balloon pump (IABP) was sited to reduce afterload and promote forward SV. Adrenaline was selected at the preference of the treating intensivist and alternative strategies are available (e.g. adding dobutamine to noradrenaline for independent manipulation of inotropy and vasoconstriction). The patient was transferred to the regional cardiothoracic centre and underwent an urgent mitral valve replacement with a good post-operative recovery.

**Fig. 4:**
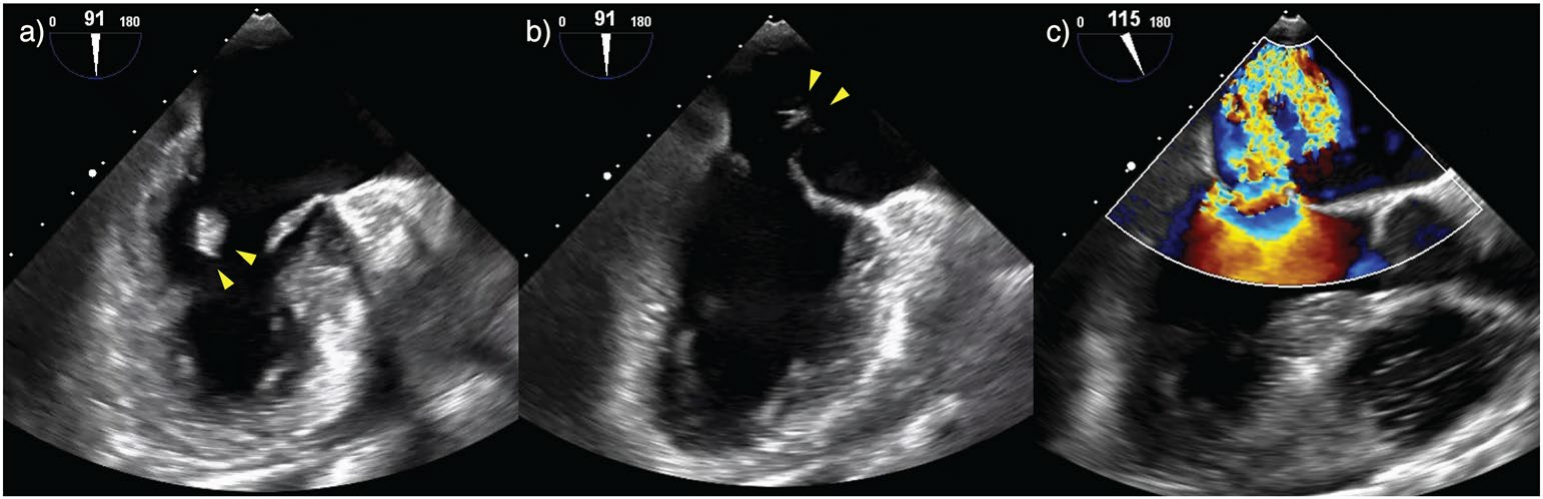
Posteromedial papillary muscle rupture. Mid-oesophageal TOE views demonstrating a ruptured posteromedial papillary muscle (arrows) in diastole (**a**) and systole (**b**) with flail leaflet and severe eccentric mitral regurgitation (**c**)

Whilst this vignette showcases acute MR in infective endocarditis, similar principles may be applied for other causes of acute MR. The most notable example of this is acute ischaemic MR due to ruptured papillary muscle which commonly presents with severe shock in the context of recent myocardial infarction (Fig. 4) (Additional file 2: Figure 5 Video—Papillary Muscle Rupture). Echo will demonstrate a rapidly equalising, often eccentrically directed MR jet, with associated regional wall motion abnormalities. This requires management strategies aimed at augmenting contractility/heart rate (inotropic/chronotropic agents), reducing afterload (inodilators/IABP insertion) and urgent referral for surgical intervention [2, 26]. The IABP provides a rescue strategy for afterload reduction when haemodynamic instability limits the use of vasodilators [43]. VA ECMO and Impella devices are emerging alternatives to provide temporary mechanical support as a bridge to surgical intervention in acute MR [44, 45].

### Clinical phenotype 2—rapid escalation in vasopressor requirements ± pulmonary oedema

Case 2 describes a progressive escalation in noradrenaline requirement and worsening oxygenation in the immediate post-operative period. The patient was managed with a conservative fluid strategy and early vasopressors due to the presumption of a mixed distributive (septic) and cardiogenic shock with early pulmonary infiltrates and hypoxaemia. A deteriorating trajectory prompted clinicians to consider second-line cardiovascular support agents with a clinical consensus favouring inotropy over further vasoconstriction due to pulmonary oedema and cool peripheries. Diuresis to achieve a negative fluid balance was also planned to limit worsening hypoxaemia and avoid re-intubation.

**Fig. 5:**
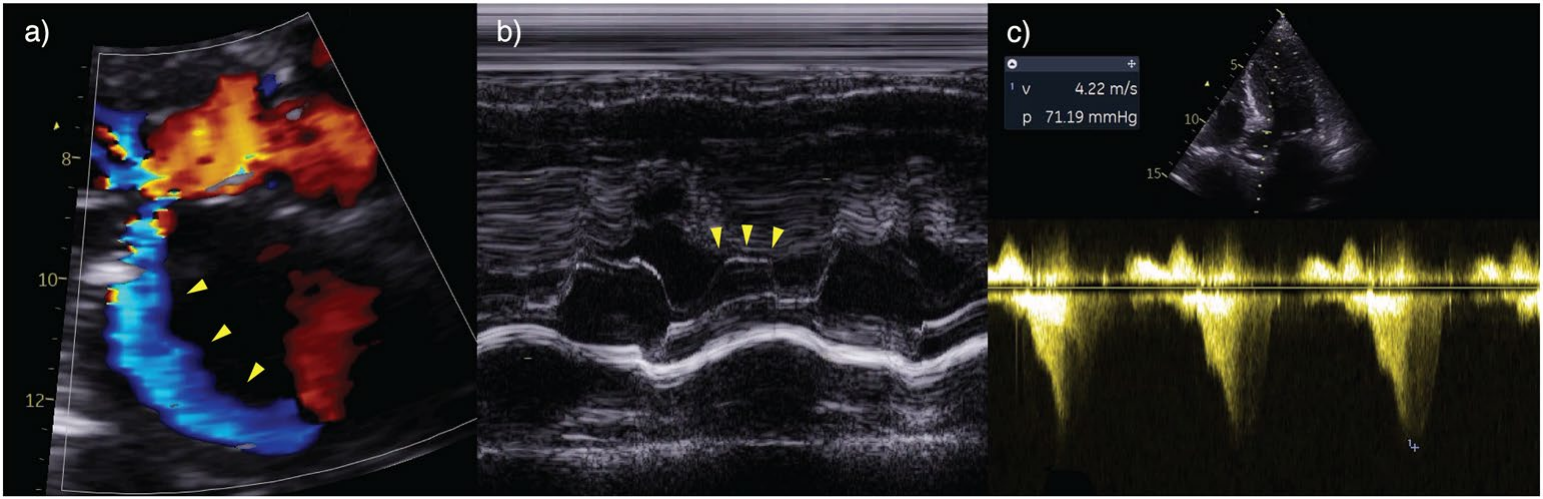
Case 2—LVOT obstruction. **a** Posteriorly directed MR jet (arrows) in the parasternal long-axis view; **b** M-mode demonstrating systolic anterior motion of the mitral valve (arrows); **c** spectral Doppler showing dynamic LVOT obstruction with a peak gradient of 71 mmHg

A TTE was performed prior to initiating these measures which demonstrated a hyperdynamic, hypertrophied left ventricle with systolic anterior motion (SAM) of the mitral valve, dynamic LV outflow tract (LVOT) obstruction and posteriorly directed MR (Fig. 5) (Additional file 3: Figure 6 Video—Case 2—LVOT Obstruction). The planned interventions of diuresis and inotropy were likely to paradoxically worsen cardiogenic shock and acute pulmonary oedema in the setting of LVOT obstruction. Careful aliquots of fluid were administered to increase preload, LV and LVOT diameter, preventing SAM and led to improved haemodynamic and respiratory status.

LVOT obstruction is common in the critically unwell [46]. SAM is caused by a combination of ‘drag’ phenomenon of the mitral valve apparatus and Venturi forces [47, 48]. Hypertrophic cardiomyopathy and hypertensive heart disease (particularly those with a ‘sigmoid’ basal septum) reduce the area of the LVOT and are predisposing risk factors for SAM. Mitral valve abnormalities, e.g. redundant anterior or posterior leaflet, papillary muscle displacement and anterior MV displacement, increase the forces acting to draw the valve towards the interventricular septum. Factors enhancing global or regional LV contractility such as distributive shock (particularly with volume depletion), Takotsubo cardiomyopathy or LAD infarction (both potentially leading to apical akinesis and hyperkinetic basal segments) may also cause LVOT obstruction [1, 46, 49–51]. Lastly, deviation of the interventricular septum towards the LV cavity, as is seen in acute cor pulmonale, predisposes individuals to SAM [52, 53].

This case emphasises that conventional management strategies applied to refractory hypoxaemia or shock (e.g. diuresis or inotropy) can worsen clinical parameters and should prompt consideration of an urgent comprehensive echo. Administration of fluids, pure vasoconstrictors (e.g. vasopressin/phenylephrine) and short-acting, titratable beta-blockade (e.g. esmolol), alongside *discontinuation* of inotropic agents and afterload reducing agents (including IABP), have all been demonstrated to reduce the incidence of LVOT obstruction [1, 46].

### Clinical phenotype 3—unilateral pulmonary oedema

Unilateral pulmonary oedema (UPO) is a hallmark feature of severe eccentric MR. Nevertheless, UPO is frequently mistaken for pneumonia, particularly in cases with diagnostic uncertainty [54].

Case 3 describes a 43-year-old with a history of myocardial infarction due to RCA occlusion on a background of antiphospholipid syndrome. Previous imaging demonstrated inferior/inferolateral regional wall motion abnormalities with mild, posteriorly directed MR. He presented with a 2-day history of productive cough, fevers and shortness of breath that was treated as severe pneumonia. The absence of ECG changes did not suggest a primary cardiac aetiology for respiratory failure despite a mild rise in cardiac biomarkers.

**Fig. 6:**
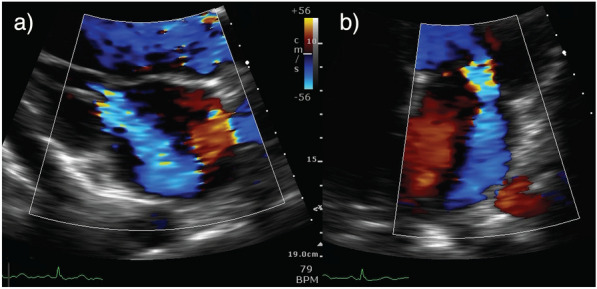
Case 3—Acute on chronic MR. **a** Posteriorly directed MR jet on parasternal long-axis (PLAX) view; **b** MR jet on apical 4 chamber (A4C) view.

Worsening oxygenation prompted referral to ICU. TTE demonstrated severe and posteriorly directed MR due to ischaemic tethering of the PMVL (Fig. 6) (Additional file 4: Figure 7 Video—Case 3—Acute on Chronic MR). His condition improved rapidly with diuresis and continuous positive airway pressure (CPAP) obviating the need for intubation.

This case demonstrates how chronic MR can become physiologically significant during acute illness [55]. Conventional therapies, in this case fluid administration, can cause inadvertent worsening of physiological parameters. Whilst comprehensive echo does not eradicate diagnostic uncertainty, findings may prompt clinicians of potential alternative or contributory diagnoses to tailor and refine treatment strategies. [35]

### Clinical phenotype 4—failure to wean from mechanical ventilation

**Fig. 7:**
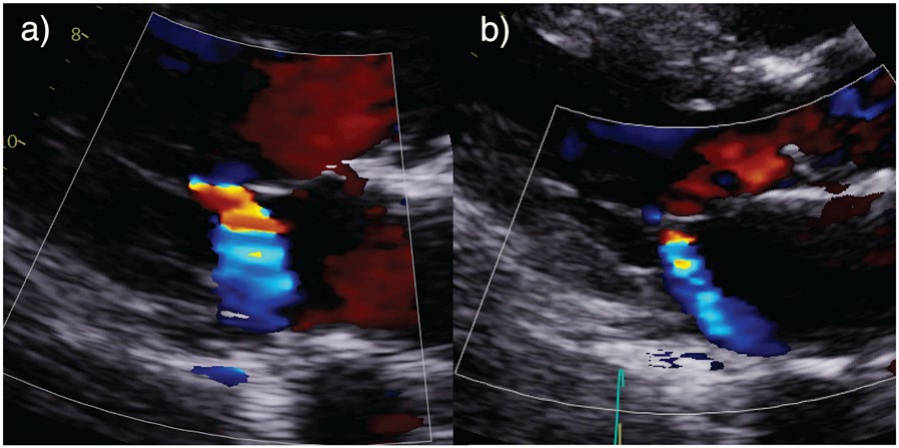
Case 4—Failure to wean from mechanical ventilation. Reduction in MR severity between **a** (initial weaning study) and **b** following diuresis and afterload reduction

Failure to wean from mechanical ventilation, defined as failure of a spontaneous breathing trial (SBT) or requirement for reintubation within 48 h of extubation, is common in the ICU [3, 56]. Case 4 describes a 76-year-old female with pneumocystis jirovecii pneumonia requiring invasive mechanical ventilation. Two SBTs resulted in tachycardia, hypertension and progressive tachypnoea with rising FiO2 requirements and bilateral pulmonary infiltrates. Repeat TTE demonstrated that the previously identified mild MR had worsened during the SBT and had become severe and posteriorly directed (Fig. 7). The patient was diuresed, commenced upon titratable afterload reducing medications and was successfully extubated directly to CPAP non-invasive ventilation.

Cardiovascular disease (including LV systolic dysfunction, diastolic dysfunction and MR) is an important cause of failure to wean and is commonly under-recognised in critical illness [57–60]. In patients situated high on the Frank–Starling curve, positive pressure ventilation reduces preload and LV transmural pressure (LV afterload). The afterload reduction promotes LV ejection, cardiac output and reduces the severity of severe MR [61, 62].

A SBT increases preload and afterload due to a reduction in intrathoracic pressure. This is compounded by increased work of breathing, adrenergic tone and myocardial oxygen demand. SBTs have been demonstrated to increase the severity of MR particularly in the context of LV systolic dysfunction.[3, 55, 60]. An echo performed immediately prior to, and during the SBT can change management in this patient group. Lung ultrasound assessing presence and number of B-lines can also help elucidate severity of interstitial pulmonary fluid [63]. Worsening MR should prompt consideration of diuresis to reduce preload and vasodilators to reduce blood pressure and afterload [3]. These measures aim to decrease regurgitation fraction and promote forward flow. Careful monitoring of loading conditions with pre-emptive use of non-invasive ventilation after extubation serves to reduce the risk of weaning failure [64]. Rarely, percutaneous or surgical intervention for MR may be required to facilitate weaning from mechanical ventilation [65].

## Assessment with echocardiography: tips and tricks

After considering the *mechanism* of the MR, two further questions are required to tailor management:

- What is the *severity* of the MR?
- What are the *physiological* *consequences* of the MR? (e.g. on LV/pulmonary haemodynamics and venous return)

### Training level in critical care echocardiography

As critical care echocardiography (CCE) expands with more intensivists acquiring advanced echo qualifications, an understanding of the basic tenets behind comprehensive techniques will be of value to intensivists and trainees. Accordingly, CCE accreditation pathways have integrated such techniques into their curriculum [66, 67]. This is especially important as management decisions at the bedside are based upon MR severity and mechanism and require integration of echo findings with clinical history, examination and haemodynamics.

Whilst not all intensivists will need to conduct comprehensive mitral valve assessment, an understanding of the overarching principles of *how* severity is determined (including the dynamic nature of such measures and their potential pitfalls), the implication of the mechanism and exacerbating conditions will greatly assist in communication and patient management. For example, transfer of information such as ‘severe MR’ is less helpful to intensivists than *acute* *severe* *MR* *due* *to* *mitral* *valve* *flail* or *acute* *on* *chronic* *severe* *MR* *due* *to* *LV* *dysfunction*.

Focused CCE (‘level 1’) has become increasingly established as a necessary skill for intensivists and is a mandatory component for ICU training in several countries [68, 69] Focused CCE has an important role in the bedside detection of significant 2D abnormalities and severe regurgitant colour jets and should prompt referral for urgent comprehensive valvular assessment [66, 68, 70]. Advanced CCE (‘level 2’) requires the clinician to be adequately trained in comprehensive measures of MR severity (using TTE ± TOE) including detailed 2D assessment, chamber size, colour jet analysis (including vena contracta, jet area and flow convergence) and spectral Doppler assessment (e.g. pulmonary vein flow, mitral inflow/regurgitant jet analysis and estimation of pulmonary pressure). Despite their inclusion in comprehensive CCE training, volumetric and semi-quantitative measures (e.g. proximal isovelocity surface area) are less applicable in the critically ill than in the outpatient setting. Expert CCE (‘level 3’) provides supervision and review of imaging performed by level 1 and 2 clinicians and apply specialised techniques including 3D assessment and procedural echo [66, 68].

### Is the MR severe?

Determining the severity of MR using comprehensive CCE requires the use of multimodal echo techniques including 2D, colour Doppler, pulsed wave Doppler (PWD) and continuous wave Doppler (CWD). Errors with quantitative measures can occur in the critically ill, particularly given challenging imaging, however this should not preclude measurement [16]. A summary of the severity measurements for MR and pitfalls are listed in Table 2.

**Table 2:** American Society of Echocardiography criteria for MR severity and potential pitfalls [27]

	MR severity			Pitfalls
	Mild	Moderate	Severe	
MV morphology	None/mild leaflet abnormality	Moderate leaflet abnormality or moderate tenting	Severe valve lesions	-Image quality often limited by mechanical ventilation and ability to optimise patient position -Requires low threshold for TOE if concerns for structural abnormalities
LV size and function LA size	Usually normal	Normal or mildly dilated	Dilated An LV ejection fraction of $\le$60% and LVESD of $\ge$40 mm is indicative of LV decompensation [71]	- Poor endocardial definition in the critical care population - Limited access to contrast agents for more accurate measurement - May not be dilated in acute severe MR - The 60/40 rule (LVEF $\le$ 60% and LVESD > 40 mm) is often used to guide surgical intervention in chronic severe MR
Colour jet area	Small, central, narrow, brief	Variable	Large central jet (> 50% of LA) or eccentric jet	- Imprecise, particularly in eccentric, wall-hugging jets - Load dependent, jet size very dependent upon systolic BP - Overestimates when MR not holosystolic - Underestimated in acute severe MR due to low MR driving pressure (due to hypotension and very high LAP)
Flow convergence	Not visible, transient or small	Intermediate in size and duration	Large throughout systole	- Problematic with multiple jets - Eccentric and constrained jets often underestimated - Non-hemispheric shape, particularly in secondary MR - Overestimates when MR not holosystolic
CWD jet	Faint, partial or parabolic	Dense but partial or parabolic	Holosystolic, dense or triangular	- Qualitative - Angle dependent—central jets appear denser than eccentric jets of higher severity - Density is gain dependent
Vena contracta (cm)	< 0.3	Intermediate	≥ 0.7	- Problematic with multiple jets - Convergence zone needed for accurate measurement - Non-hemispheric shape, particularly in secondary MR
Pulmonary vein flow	Systolic dominance	Normal or systolic blunting	Systolic flow reversal or minimal systolic flow	- MR may affect flow pattern in individual PVs - Challenging to image with TTE - Systolic blunting not specific for MR—also elevated LAP, AF - All pulmonary veins must be assessed when using TOE, particularly in the presence of eccentric MR jets
Mitral inflow	A-wave dominant	Variable	E-wave dominant (> 1.2 m/s)	- Not specific to MR - Affected by severe LV dysfunction (low E wave even in severe MR)
Effective regurgitant orifice area (EROA), 2D proximal isovelocity surface area (PISA) (cm^2^)	< 0.20	0.2–0.39	≥ 0.40	- Less accurate with eccentric/multiple jets - Non-hemispheric shape, particularly in secondary MR. 3D PISA may improve accuracy but is seldom performed in the ICU - Significant amplification of small measurement errors through step-wise calculations
RVol (ml)	< 30	30–59	≥ 60	- Significant amplification of small measurement errors through step-wise calculations - Not valid if coexistent AR - Volumetric vs PWD methods may give different results
RF (%)	< 30	30–49	≥ 50	

MV, mitral valve; LV, left ventricle; LA, left atrium; CWD, continuous wave Doppler; TTE, transthoracic echocardiography; TOE, transoesophageal echocardiography; LVESD, LV end-systolic diameter; LAP, left atrial pressure; AF, atrial fibrillation; EROA, effective regurgitant orifice area; PISA, proximal isovelocity surface area; RVol, regurgitant volume; RF, regurgitant fraction; AR, aortic regurgitation; PWD, pulse wave Doppler

#### 2D assessment

2D imaging (using conventional and zoomed views) is the key initial step to ‘fan’ through the valve and assess for clear structural abnormalities or coaptation defects (e.g. prolapse, flail, gap between leaflet tips) that would be suggestive of severe MR. LV and LA size also provide clues as to the acuity of regurgitation.

TOE provides superior resolution of the MV anatomy and clinicians should have a low threshold in cases of diagnostic or mechanistic uncertainty. This is particularly important with prosthetic mitral valves which are frequently challenging to image with TTE [72]. Assessment of prosthetic valves is beyond the scope of this article.

Newer 3D transducers facilitate multiplane reconstruction of the mitral valve which may be rotated to achieve a “surgical view” from the perspective of the left atrium. This allows simultaneous evaluation of the structure and function of the valve and subvalvular apparatus and has gained popularity in directing percutaneous and surgical intervention. 3D should be considered complementary to systematic 2D interrogation of the valve and relies upon good image resolution for image generation.

#### Colour Doppler

**Fig. 8:**
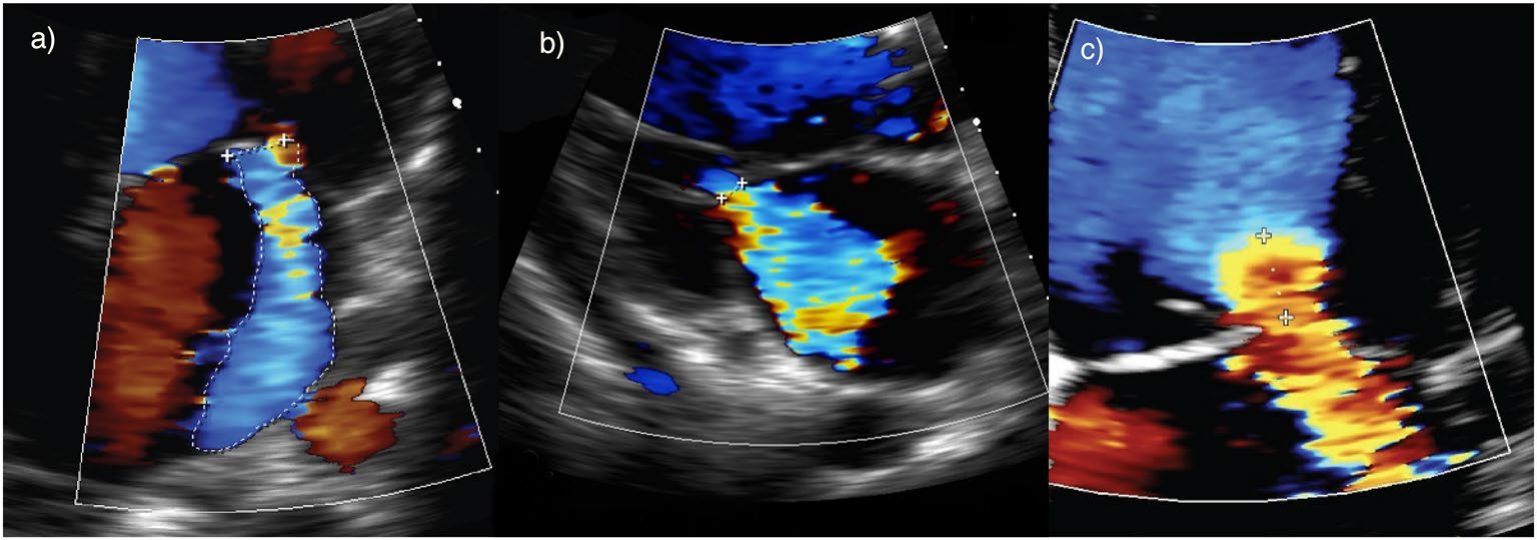
Jet area, vena contracta and PISA. **a** MR jet area measurement; **b** vena contracta measurement and **c** PISA measurement

There are three components to a MR jet: the flow convergence proximal to the valve, the vena contracta (smallest jet diameter) and the jet area (Fig. 8). All three of these parameters can be measured to assess severity. In addition, colour Doppler helps to determine the optimal angle of interrogation for PWD and CWD (off-axis imaging may be required).

##### MR jet area

The ‘colour flow jet area’ and ‘jet area: LA area ratio’ are standard parameters to determine MR severity, with >10 cm^2^ and >50%, respectively, considered to be severe. Particularly in the critically ill, there is an unpredictable relationship between jet area and severity and the American Society of Echocardiography (ASE) recommend *against* its use for severity assessment [27, 73]. Increased driving pressure across the valve (e.g. raised LVEDP, fluid overload) will increase the jet area for a given regurgitant orifice area [38]. Conversely, systemic hypotension will reduce the driving pressure across the valve and thus underestimate MR severity. Eccentric, wall-hugging jets will appear considerably smaller than non-constrained central jets due to the Coanda effect. The presence of an abnormal, horizontal ‘splay’ signal of colour Doppler along the atrial surface of the valve may be the only feature of severe MR in an otherwise benign or equivocal looking jet [74].

##### Vena contracta

The vena contracta (VC) is the narrowest portion of the jet with the highest velocity—located immediately downstream of the regurgitant orifice area. VC is less load dependent that other measures and therefore may be particularly useful in critical care. VC diameter should be measured using a zoomed view (often the parasternal long-axis view), a narrow colour box (to optimise temporal resolution) and with jet direction perpendicular to the insonation beam (applying axial measurement accuracy) [12]. 2D measurement of VC diameter relies upon the concept that the regurgitant orifice area is circular. Whilst this is mostly the case in *primary* *MR*, the regurgitant orifice area is more elongated along the coaptation line in *secondary* *MR,* meaning VC diameter may both overestimate or underestimate MR severity in the same patient depending upon the imaging plane [75, 76]. The flow convergence zone of the regurgitant jet should be visualised for accurate measurement of the VC. It is not recommended to add the VC of multiple jets together to determine a *total* *VC* if multiple orifices exist [27, 38].

##### Flow convergence (proximal isovelocity surface area; PISA) and EROA

As blood flow converges towards a regurgitant orifice, it forms concentric ‘shells’ of increasing velocity and decreasing surface area. The presence of a flow convergence zone is likely to indicate moderate or severe MR even in the absence of a significant colour jet when using the recommended Nyquist limit range (50–70 cm/s). Manipulation of the colour scale baseline enables visualisation of an aliasing threshold and measurement of the *proximal* *isovelocity* *surface* *area* (PISA): from VC to point of colour aliasing. Numerous assumptions (e.g. PISA assumed to be hemispherical), measurement error and amplification of errors through step-wise calculation lessens the accuracy of PISA and therefore is used less frequently in the critically ill [38]. Accuracy may be improved by applying only in central MR jets and using TOE. If performed, the effective regurgitant orifice area (EROA) can be calculated and considered severe if >0.4 cm^2^. The EROA is subsequently multiplied by the MR velocity time integral (VTI) to calculate the regurgitant volume (RVol) [12, 28].

PISA has become the preferred technique for quantification of EROA and RVol in the outpatient setting but is limited by numerous assumptions and prone to significant error. In the critically ill, where specific volumes are dynamic and conditions suboptimal, PISA is arguably less applicable and should be applied with caution and only in combination with other parameters.

#### Pulse wave Doppler

PWD assessment of flow through the mitral valve provides supporting evidence of MR severity. This is represented by an elevated E wave velocity (≥1.5 m/s). These are non-specific findings and may be seen in many conditions including mitral stenosis, diastolic dysfunction/restrictive heart disease, hyperdynamic circulation or tachycardia [77]. Similarly, E wave velocity is often low (<1 m/s) in the setting of severe LV dysfunction even if significant MR is present. The rise in MV inflow velocity in MR occurs independently of the diastolic function. Severe MR therefore negates the use of MV inflow velocity and E/e’ as a marker of LVEDP and diastolic dysfunction [32, 77].

Flow characteristics within the pulmonary veins is the most important PWD parameter to determine MR severity. This is discussed in more detail in the section on physiological consequences of MR.

#### Continuous wave Doppler

The density of CWD trace provides qualitative evidence of MR severity. As severity increases, a greater quantity of blood flows through the regurgitant orifice—represented by an increasingly dense CWD signal. The angle dependency of CWD is a potential pitfall to this and central jets may appear denser than more severe eccentric jets due to malalignment. Similarly, density is gain dependent, meaning users should take care to avoid excessive gain when optimising the spectral Doppler trace.

**Fig. 9:**
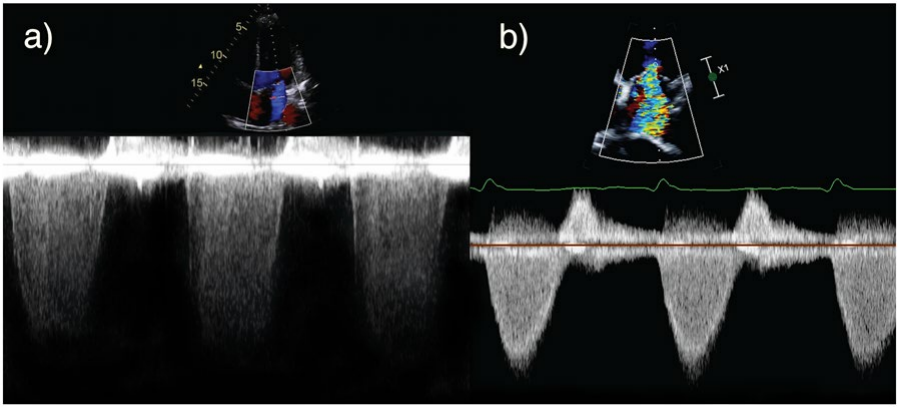
Continuous wave Doppler. Incomplete, parabolic Doppler signal in mild MR (**a**) versus dense, triangular shaped Doppler signal in severe MR (**b**)

The shape of the MR jet is also important. A dense, holosystolic, triangular shaped CWD trace is indicative of raised LAP or significant regurgitant flow (Fig. 9) and is analogous to a large V wave on a pressure waveform. Many of the aforementioned techniques will either over- or under-estimate MR severity if MR is not holosystolic, e.g. in mitral valve prolapse (late systolic), severe LV dysfunction (biphasic) or diastolic MR [27]. Diastolic MR is likely to be under-recognised in the critically ill particularly given the frequency of atrioventricular conduction abnormalities [27, 78, 79].

CWD analysis of the TR jet is useful for the identification of raised pulmonary artery pressure as a consequence of MR.

#### Additional volumetric measures of MR

An alternative method for calculating regurgitant volume is by subtracting the LV SV (calculated using LVOT area and PWD LVOT VTI) from the Simpson’s biplane-derived stroke volume or using PWD technique at the mitral annulus. Simpson’s biplane is known to underestimate LV volumes compared with contrast echo, 3D echo and cardiac MRI and may lead to errors that are compounded in subsequent calculations. Volumetric calculations are challenging and less applicable to the critical care population.

### Physiological consequences of MR

The intensivist should consider the upstream and downstream consequences of MR to be of equal importance to the regurgitation severity. Chronic MR leads to dilation and volume overload of the LA and LV, causing elevated mean LAP that is transmitted to the pulmonary venous circulation. With time, LA hypertension leads to remodelling, fibrosis and reduced compliance and is an important cause of post-capillary pulmonary hypertension (PHT). Ultimately this may lead to right ventricular remodelling and failure. Assessment of PHT and the RV is beyond the scope of this paper and the reader is directed elsewhere [80, 81]. In acute MR, the LA is unable to acutely dilate to accommodate an increased volume precipitating a rapid elevation in LAP and pulmonary pressures.

**Fig. 10:**
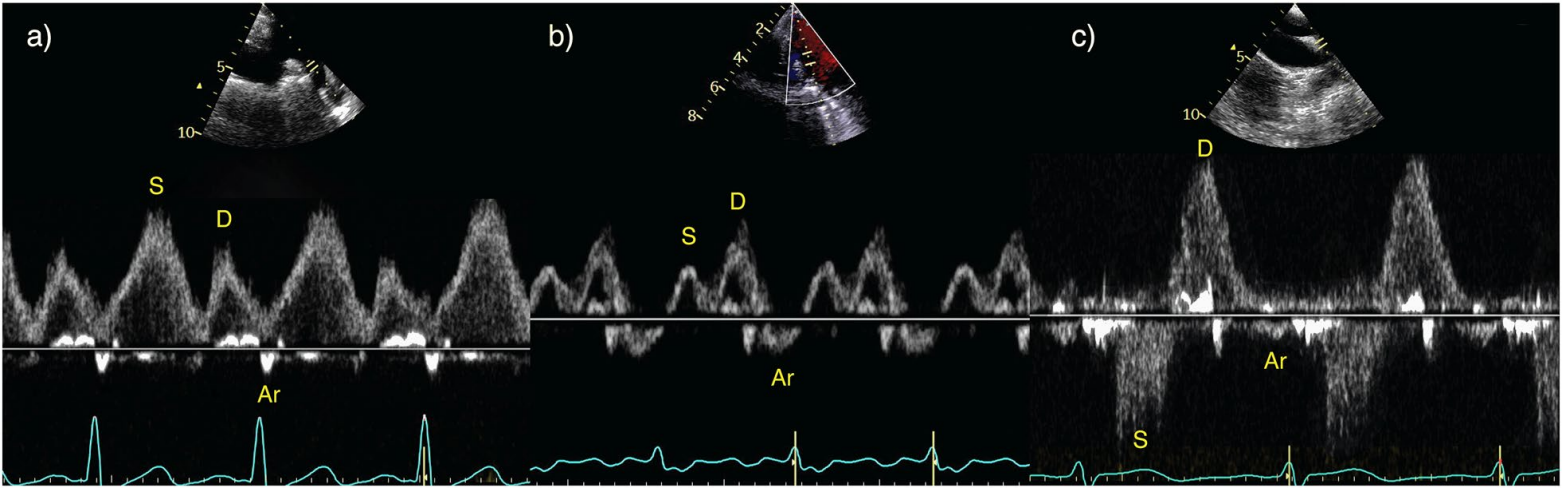
Pulmonary venous flow patterns. **a** Normal systolic dominant flow pattern with a low velocity and short duration of flow reversal during atrial systole (Ar). **b** Blunting of systolic flow (S) with dominant diastolic flow (D) and a higher velocity and increased duration of Ar, indicative of raised left atrial pressure. **c** Systolic flow reversal (S) suggestive of severe MR or severely elevated left atrial pressure

PWD of the pulmonary venous flow provides a useful adjunctive measure of MR severity. This is performed by placing PWD within one of the right pulmonary veins in the far field of the A4C view on TTE [12, 82]. It is possible to assess all pulmonary veins when using TOE; an omniplane angle of 110° can locate left-sided veins and 60–80° used for right sided veins. A minimum TOE dataset should always include assessment of all pulmonary veins where possible. Increasing MR severity reduces the systolic component of pulmonary venous flow and eventually leads to systolic flow reversal (Fig. 10). Technical difficulty and limited image quality unfortunately renders this assessment challenging using TTE within the ICU [83]. Furthermore, recommendations suggest more than one pulmonary vein should be assessed in MR due to the possibility of the MR jet selectively entering a single vein [12, 28]. This is usually only achievable with TOE.

Pulmonary venous flow alterations are not specific for mitral valve disease and are seen in other causes of raised LAP. In addition to the conventional chronic causes of raised LAP (e.g. diastolic dysfunction, mitral stenosis), considerations in the critically unwell include any cause of volume overload or acute systolic/diastolic dysfunction (e.g. septic cardiomyopathy) [83, 84]. A reduced IVRT of <60 ms and mitral ‘L’ wave of >20 cm/s are supporting features of raised LAP but are beyond the scope of this article [32]. The reader is directed elsewhere for excellent review articles of the echo assessment of LAP [32, 83, 84].

Despite its lack of sensitivity for MR, the presence of blunted systolic pulmonary venous flow provides the clinician with a window into the patient’s cardiopulmonary physiology. The ageing critical care population with an increased incidence of degenerative mitral valve disease and functional MR is leading to the frequent coexistence of multiple causes of raised LAP (e.g. MV calcification with functional MR, alongside LV hypertrophy and diastolic dysfunction). These complementary factors may lower the threshold for decompensation during acute illness. Fortunately, many management principles in these conditions overlap (e.g. diuresis) allowing clinicians to use the upstream and downstream consequences to guide ongoing management.

As previously mentioned, acute MR may lead to the preservation of EF even in the setting of profound shock. Doppler-derived assessment of LV stroke volume is a simple technique that should be within the armamentarium of all critical care sonographers and integrated into conventional assessments of LV function. Peak velocity of the MR CWD signal also provides indirect evidence of haemodynamic compromise. Maximum peak MR velocity is usually between 4–6 m/s due to the high systolic gradient between LV and LA. Low peak MR velocity (less than 4 m/s) is suggestive of a reduced gradient due to hypotension and raised LAP [27].

## Take home points for MR in critical care

1. Serial assessment is crucial and allows recognition of physiologically significant MR and the development of tailored management plans (e.g. diuresis, avoiding bradycardia and hypertension, extubation directly to CPAP).
2. Identifying the mechanism of MR is of equal importance as the severity. Severe structural abnormalities should prompt referral for definitive intervention (e.g. MV replacement/repair). Early implementation of mechanical haemodynamic interventions (IABP, Impella) to stabilise physiology as a bridge to intervention should be considered.
3. Secondary MR is likely to be more common in the ICU. Critical illness will lower the threshold for decompensation and early imaging can recognise and limit iatrogenic worsening of MR.
4. Quantitative parameters of MR severity are limited by issues with precision, reproducibility and amplification of small measurement errors. Provided the pitfalls of these measures are considered, quantification of MR is useful for serial assessment and provides a common language during specialist referral.
5. The presence of a flow convergence zone at the recommended Nyquist limit range of 50–70 cm/s is likely to indicate moderate or severe MR even in the absence of a significant colour jet.
6. The upstream and downstream consequences of MR, including reduced cardiac output, pulmonary venous hypertension and RV dysfunction, are important to recognise and quantify in the critically ill patient.
7. TOE provides superior resolution of mitral valve anatomy and clinicians should have a low threshold in cases of diagnostic or mechanistic uncertainty. This is particularly important with prosthetic mitral valves [72].

## Conclusions

Critical care echo is an expanding subspecialty that allows recognition and management of MR during acute illness. The intensivist is uniquely positioned to apply advanced valvular assessment at the bedside to optimise cardiorespiratory physiology and identify patients requiring percutaneous or surgical intervention. MR is dynamic and repeated assessment is key. Comprehensive assessment in the ICU should integrate loading factors and clearly describe the mechanism, severity and the upstream/downstream complications.

## Supplementary Information

**Additional file 1.** Videos to accompany images for Case 1—Infective Endocarditis with severe mitral regurgitation.

**Additional file 2.** Videos to accompany images for Figure 5—Papillary Muscle Rupture.

**Additional file 3.** Videos to accompany images for Figure 6—LVOT Obstruction.

**Additional file 4.** Videos to accompany images for Figure 7—Acute on Chronic MR.

## Data Availability

Not applicable.

## Abbreviations

A4C: Apical four chamber view
ACEI: ACE inhibitors
AF: Atrial fibrillation
ALPM: Anterolateral papillary muscle
AMVL: Anterior mitral valve leaflet
AR: Aortic regurgitation
ARBs: Angiotensin receptor blockers
ARDS: Acute respiratory distress syndrome
ASE: American Society of Echocardiography
AV: Aortic valve
CCBs: Calcium channel blockers
CM: Cardiomyopathy
CWD: Continuous wave Doppler
ECG: Electrocardiogram
Echo: Echocardiography
ED: Emergency department
EROA: Effective regurgitant orifice area
FiO2: Fraction of inspired oxygen
HCM: Hypertrophic cardiomyopathy
IABP: Intra-aortic balloon pump
IPPV: Invasive positive pressure ventilation
IVRT: Isovolumic relaxation time
LA: Left atrium
LAA: Left atrial appendage
LAD: Left anterior descending coronary artery
LAP: Left atrial pressure
LV: Left ventricle
LVEDP: LV end-diastolic pressure
LVH: Left ventricle hypertrophy
MR: Mitral regurgitation
MS: Mitral stenosis
PE: Pulmonary embolism
PEEP: Positive end expiratory pressure
PHT: Pulmonary hypertension
PISA: Proximal isovelocity surface area
PLAX: Parasternal long-axis view
PMPM: Posteromedial papillary muscle
PMVL: Posterior mitral valve leaflet
PWD: Pulse wave Doppler
RCA: Right coronary artery
RF: Regurgitant fraction
RVol: Regurgitant volume
SAM: Systolic anterior motion of the mitral valve
SBT: Spontaneous breathing trial
SV: Stroke volume
SVR: Systemic vascular resistance
TOE: Transoesophageal echocardiogram
TEE: Transesophageal echocardiogram
TR: Tricuspid regurgitation
TTE: Transthoracic echocardiogram
UPO: Unilateral pulmonary oedema
VC: Vena contracta
VTI: Velocity time integral
